# Progressive Multifocal Leukoencephalopathy Mimicking Stroke in a Patient With Newly Diagnosed HIV: A Case Report

**DOI:** 10.7759/cureus.97303

**Published:** 2025-11-20

**Authors:** Thaw Hlaing, Thet Maung

**Affiliations:** 1 Internal Medicine, Sandwell and West Birmingham Hospitals NHS Trust, Birmingham, GBR

**Keywords:** human immunodeficiency virus, john cunningham virus, progressive multifocal leukoencephalopathy, stroke mimic, white matter lesion

## Abstract

Progressive multifocal leukoencephalopathy (PML) is a rare but often fatal demyelinating disease of the central nervous system (CNS) caused by reactivation of the John Cunningham (JC) virus in immunocompromised individuals, most commonly those with advanced HIV infection. We report the case of a 56-year-old woman initially investigated for possible ovarian malignancy who presented with progressive left-sided weakness, mimicking ischemic stroke. However, the discordance between the clinical presentation and radiological findings prompted multidisciplinary evaluation, which led to the detection of previously unrecognized HIV infection. Subsequent cerebrospinal fluid (CSF) analysis confirmed the presence of JC virus, establishing the diagnosis of progressive multifocal leukoencephalopathy. Despite prompt initiation of antiretroviral therapy, the patient further deteriorated, culminating in death. This case highlights the diagnostic complexity of PML and the importance of considering HIV-related opportunistic infections in atypical neurological presentations.

## Introduction

Progressive multifocal leukoencephalopathy (PML) is a rare but often fatal demyelinating disease of the central nervous system (CNS) caused by reactivation of the John Cunningham (JC) virus in immunocompromised individuals [1,2]. First described in 1958, PML classically occurs in patients with advanced HIV infection but has increasingly been recognized in association with other immunosuppressive conditions, including hematological malignancies, autoimmune diseases, and the use of monoclonal antibody therapies such as natalizumab, rituximab, and efalizumab. The overall incidence of PML is estimated at approximately 0.2 cases per 100,000 person-years, although this varies considerably depending on the underlying risk population [3].

The JC virus is a ubiquitous polyomavirus that remains latent in the kidneys and lymphoid tissues in the majority of adults [3]. Seroprevalence studies suggest that up to 70%-90% of the adult population has been exposed to the virus, yet only a small minority develops PML when cellular immunity is compromised. Reactivation typically occurs in the context of impaired cellular immunity, leading to lytic infection of oligodendrocytes and widespread demyelination. Clinically, PML presents with a diverse range of neurological deficits, commonly visual disturbances, motor weakness, cognitive decline, and speech impairment, reflecting multifocal involvement of cerebral white matter.

Despite advances in diagnostic techniques, including MRI and JC virus detection via cerebrospinal fluid (CSF), PML remains a diagnostic and therapeutic challenge due to symptoms overlapping with other neurological conditions (e.g., stroke, encephalitis, and tumors). Early recognition is crucial, as rapid restoration of immune function remains the cornerstone of management and determines prognosis.

Early initiation of antiretroviral therapy (ART) is crucial in HIV-associated PML, as immune restoration provides the potential for halting disease progression. In general, PML has a mortality rate of 30%-50% in the first few months following diagnosis. Those who survive the disease may also be left with severe neurological disabilities [3].

We present a case of PML in a 56-year-old lady with a subacute onset and progression of neurological deficit, highlighting the diagnostic challenges as symptoms were initially mimicking a stroke. Incidental finding of lymphadenopathy shifts the diagnosis toward possible cerebral lymphoma, but a new diagnosis of HIV offers a wider range of differentials, including opportunistic infections. Further workup for CSF PCR results showed JC virus, confirming PML. Despite prompt involvement of a multidisciplinary team (MDT) and highly active antiretroviral therapy initiation, the patient later developed PML-associated immune reconstitution inflammatory syndrome (IRIS), resulting in death.

## Case presentation

This patient was a 56-year-old Caucasian woman (ex-smoker) who was under investigation for suspected ovarian cancer due to recent weight loss of 2 stones, lethargy, and mildly raised cancer antigen 125 (CA-125) of 36 U/mL. She was admitted to the hospital with a two-week history of progressive weakness and numbness on the left side of the body, initially involving the left leg and then progressing to the left arm. She was independently mobile previously but became wheelchair-dependent over the course of two weeks due to progressive weakness. This was not associated with facial asymmetry or slurred speech. Clinical examination revealed left-sided visual inattention, reduced power of 2/5 in both left upper and lower limbs, and a positive Babinski sign on the left side. Motor power, sensation on the right side, and reflexes were normal. There were no facial asymmetry or cerebellar signs.

**Figure 1:**
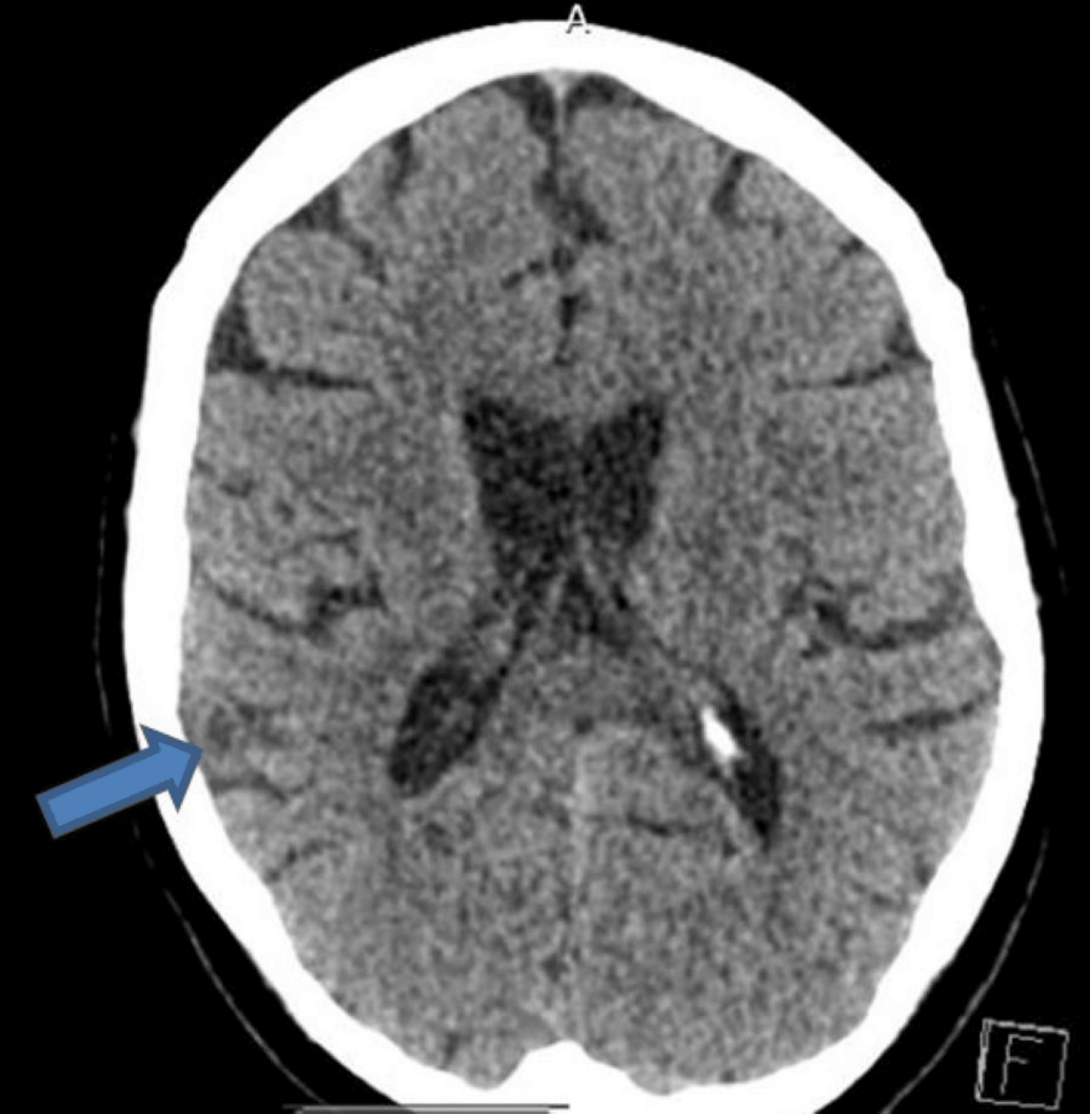
Head CT (axial view) showing right parietal lobe hypoattenuation (arrow)

The head CT on admission showed right parietal lobe hypoattenuation, concerning of subacute infarct (Figure 1). There were no features of midline shift, herniation, hydrocephalus, or pathological contrast enhancement. The patient was treated as a stroke with aspirin 300 mg, and further investigations for stroke workup, such as head MRI with contrast and US carotid Dopplers, were arranged. Thoracic, abdominal, and pelvic CT previously arranged as an outpatient as part of the investigation for ovarian cancer was arranged as an inpatient.

**Figure 2:**
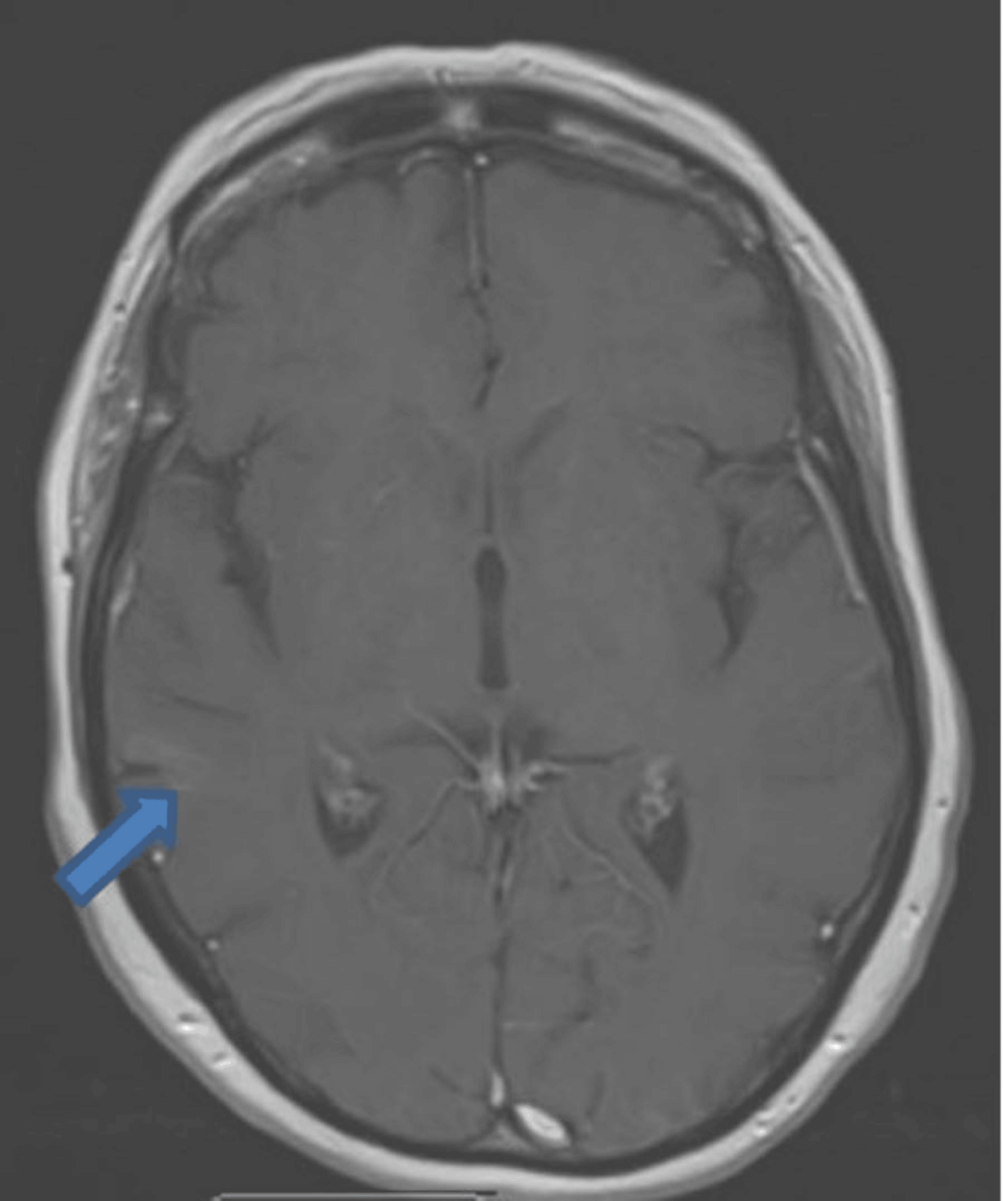
Head MRI without contrast (axial flair): faint diffusion restriction seen at the right parietal gyrus area (arrow)

Subsequent head MRI without contrast showed faint diffusion restriction seen at the right parietal gyrus area (Figure 2). This patient was discussed with the stroke team, who advised that the patient’s clinical presentation of unilateral weakness and sensory loss, more prominent in the lower limb and then progressing to the upper limb, did not correlate with the site of the infarct area noted on head CT and MRI. With the patient’s clinical presentation, the usual site of the infarct area would be the anterior cerebral artery territory, such as the medial frontal lobe, paracentral lobule, cingulate gyrus, and sometimes the corpus callosum.

Given the unusual presentation of the infarct not following the anterior cerebral artery territory and given the patient‘s background history of suspected ovarian cancer, paraneoplastic syndrome was suspected. Therefore, further investigations, including head MRI with contrast, tumor markers, and paraneoplastic antibodies, were requested, along with thoracic, abdominal, and pelvic CT. Neuroradiology MDT was involved.

**Figure 3:**
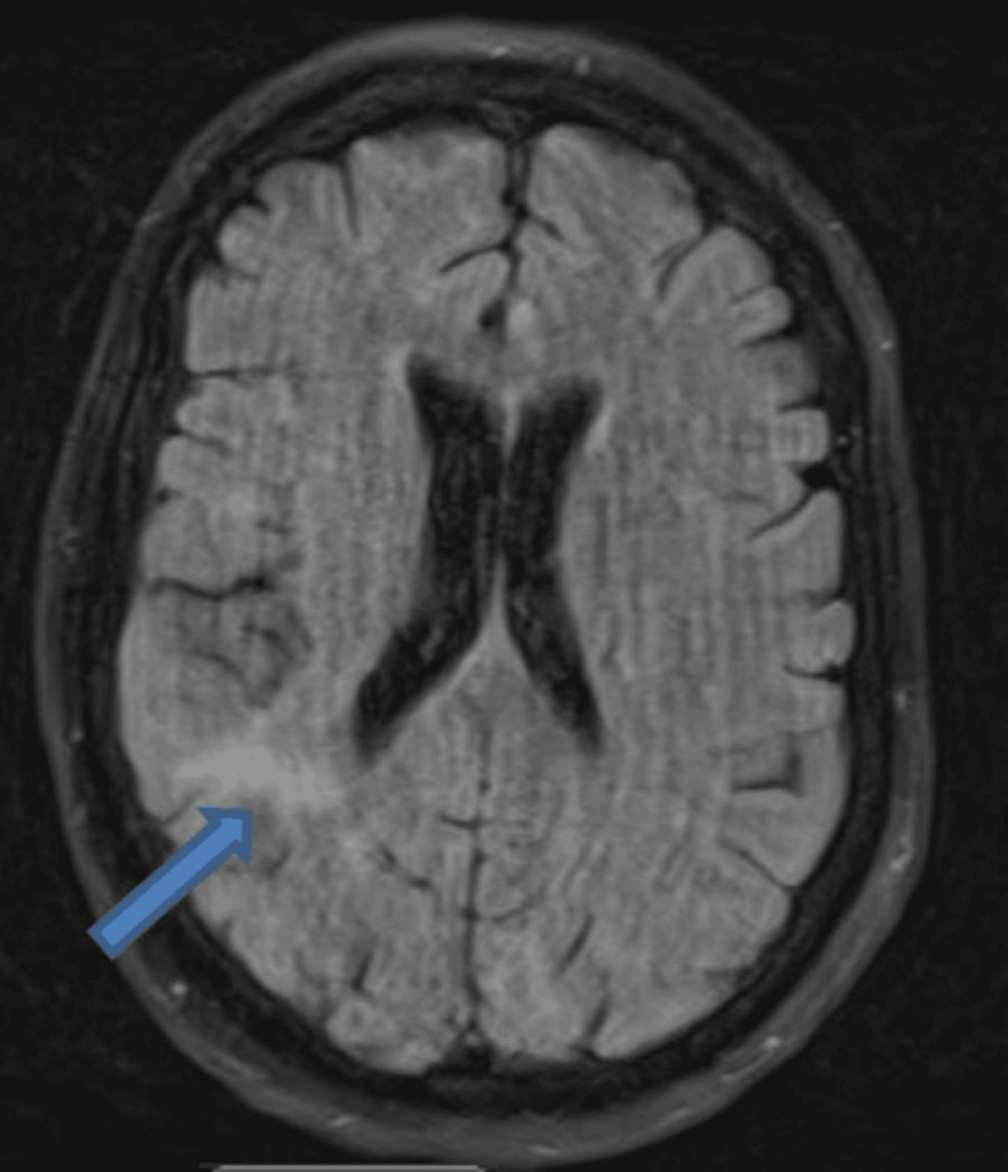
Head MRI T1 image with contrast (axial view): increasing diffusion restriction in white matter around the right parietal gyrus and parietal lobe (arrow)

Subsequent head MRI with contrast showed increasing diffusion restriction in white matter around the right parietal gyrus and parietal lobe (Figure 3) [4,5]. On thoracic, abdominal, and pelvic CT, there was no evidence of primary malignancy, but bilateral pulmonary nodules along with supraclavicular, subpectoral, and axillary lymph nodes were noted, raising the suspicion for lymphoproliferative disorder. This patient was discussed with the hematology team who has advised for further investigations such as lactate dehydrogenase (LDH), hepatitis B antigen, serum hepatitis C antibody, HIV, angiotensin-converting enzyme (ACE), TB quantiferon, blood film (on the possibility that anything malignant or clonal can be identified), lumbar puncture with CSF analysis including cytospin, lymphoproliferative disorder panel, and radiology involvement to determine the biopsy site.

Neuroradiology MDT advised that the lesion visualized on the MRI scan represents a space-occupying lesion rather than ischemia. The patient was also discussed in the neuro-oncology MDT, where the images were reviewed, and the conclusion was a diffuse lesion involving the right temporoparietal lobe on the FLAIR sequence. No abnormal contrast enhancement was seen, and it was advised to arrange the biopsy of enlarged lymph nodes to rule out lymphoproliferative disorder. A US-guided cervical lymph node biopsy was not successful because the lymph nodes are too small and not amenable for biopsy.

Unexpectedly, the patient tested positive for HIV, a finding that significantly altered the subsequent management plan. The patient was discussed with the HIV team, and a wider differential of opportunistic CNS infections, including cryptococcal meningitis, TB meningitis, varicella zoster virus (VZV) vasculitis, toxoplasmosis, and progressive multifocal leukoencephalopathy, were considered at this point. Further workups for HIV, including a repeat serum sample to confirm the result, CD4 lymphocyte count, HIV viral load, and glucose-6-phosphate dehydrogenase, were done.

After confirming the HIV-positive result with a repeat sample, the patient was commenced on highly active antiretroviral therapy (HAART), which includes Descovy (tenofovir alafenamide/emtricitabine) (25/200 mg) once a day and dolutegravir 50 mg once a day. Long-term septrin prophylaxis 480 mg once a day was also initiated.

On initial evaluation, the patient demonstrated advanced immunosuppression with a CD4 T-lymphocyte count of 43 cells/µL (4.8%) and a high HIV viral load of 139,000 copies/mL, consistent with newly diagnosed, severe HIV infection. Opportunistic infection screening revealed negative Toxoplasma IgG and IgM, negative cryptococcal antigen, and negative syphilis serology. Serology was positive for past exposure to cytomegalovirus (CMV IgG positive, IgM negative) and Epstein-Barr virus (EBV NA IgG positive, VCA IgM negative), indicating prior infection without evidence of active viral replication.

After the HIV-positive result, the patient was re-discussed in neuro-oncology MDT, and the outcome was that, in the context of HIV and MRI findings of white matter abnormality, progressive multifocal leukoencephalopathy (PML) is a possible differential, and it was advised to rule out John Cunningham (JC) virus in CSF fluid. Detection of the JC virus in the cerebrospinal fluid established the definitive diagnosis of progressive multifocal leukoencephalopathy (PML).

Lumbar puncture was done to look for opportunistic CNS infections, and JC viral DNA was detected in CSF analysis (Table 1) [6].

**Table 1:** CSF analysis with reference range CSF: cerebrospinal fluid, JC: John Cunningham

Test	Result	Reference range
Appearance	Clear color	N/A
WBC	0 cells/cumm	<5 cells/cumm
Red cell	3 cells/cumm	0 cells/cumm
Glucose	2.6 mmol/L	2.5-4.5 mmol/L
Protein	0.43 g/L	0.15-0.6 g/L
CSF culture	No organism isolated	N/A
Mycobacterium culture	Not isolated	N/A
Enterovirus RNA	Not detected	N/A
Varicella zoster DNA	Not detected	N/A
BK virus RNA	Not detected	N/A
Parechovirus RNA	Not detected	N/A
JC virus DNA	Detected	N/A

Despite initiation of HAART, the patient‘s condition further deteriorated with worsening of neurological deficits, and a repeat head CT showed progression of the disease. She was discharged to a rehabilitation bed and then to a nursing home, but was readmitted to the hospital one week after discharge and was managed for chest sepsis and progressive multifocal leukoencephalopathy-immune reconstitution inflammatory syndrome (PML-IRIS). Unfortunately, the patient further deteriorated despite antibiotics and steroids. Therefore, a supportive care pathway (SCP) was initiated, and the patient sadly passed away four months after the symptom onset.

## Discussion

This case illustrates the complex diagnostic challenge posed by an atypical neurological presentation in a patient initially being investigated for suspected ovarian malignancy, ultimately found to have HIV-associated progressive multifocal leukoencephalopathy (PML). It underscores the importance of maintaining a broad differential diagnosis when neurological deficits and radiological findings are incongruent and highlights the evolving clinical landscape of HIV-related opportunistic infections in the modern era of antiretroviral therapy (ART) [7].

The patient presented with a progressive left-sided weakness and sensory deficit evolving over two weeks, initially suggestive of an ischemic cerebrovascular event. Neuroimaging initially demonstrated right-sided subacute infarcts on CT and MRI, which led to a working diagnosis of stroke. However, the clinical presentation (predominantly lower-limb weakness, which became progressive to the upper limbs) and discordance with vascular territories prompted reconsideration of the diagnosis.

This discrepancy highlights an important clinical learning point: when imaging and clinical findings do not correlate, alternative diagnoses beyond vascular events must be considered, including inflammatory, demyelinating, neoplastic, or infectious processes. In this case, the evolving radiological features and enhancement patterns on MRI raised suspicion for a space-occupying or demyelinating lesion, which ultimately guided further investigations.

Given the patient’s background of a raised CA-125 and recent weight loss, a paraneoplastic neurological syndrome or lymphoproliferative disorder was initially suspected. Paraneoplastic syndromes such as those associated with anti-Hu, anti-Yo, anti-Ma2, anti-Ri, anti-CV2, or anti-amphiphysin antibodies can cause multifocal neurological deficits and MRI findings that resemble demyelination or infarction. However, test results for these antibodies were negative. Subsequent CT of the thorax, abdomen, and pelvis did not reveal a primary tumor, and lymph node biopsy was not feasible due to the small size of the nodes. This diagnostic uncertainty further illustrates how malignancy-associated neurological syndromes can closely mimic central nervous system infections or inflammatory demyelination.

The discovery of HIV infection with profound immunosuppression (CD4 count of 43 cells/µL) was pivotal in redirecting the diagnostic approach. HIV-associated neurological disorders can manifest in diverse ways, ranging from HIV encephalopathy to opportunistic infections and neoplasms such as primary CNS lymphoma [8]. The negative workup for toxoplasmosis, cryptococcosis, and other opportunistic infections, combined with MRI findings of asymmetric white matter lesions without contrast enhancement, supported the clinical suspicion of PML. Confirmation of JC virus DNA in the cerebrospinal fluid (CSF) established the diagnosis.

PML is a demyelinating disease of the central nervous system caused by reactivation of the John Cunningham (JC) virus, leading to multifocal demyelination of cerebral white matter [9]. Profound CD4 T-cell depletion in advanced HIV infection compromises immune surveillance, allowing uncontrolled viral replication and direct cytolytic damage to myelin-producing cells. It typically occurs in patients with severe cellular immunodeficiency. Although the incidence of PML has markedly declined since the introduction of combination ART, it remains a serious and often fatal complication in individuals with advanced HIV infection or in those presenting late in the disease course [10].

Typical MRI features include multifocal, asymmetric, subcortical white matter lesions that are hyperintense on T2-weighted and FLAIR sequences, without mass effect or significant enhancement, findings consistent with this case. Histopathological confirmation is rarely required when JC virus DNA is detected in CSF via PCR, as in this patient.

The cornerstone of PML management in HIV-infected individuals is prompt initiation of ART to restore immune function [11]. Unfortunately, immune reconstitution may paradoxically trigger PML-associated immune reconstitution inflammatory syndrome (PML-IRIS), leading to clinical deterioration, as observed in this case [12]. Despite appropriate initiation of HAART and supportive management, the patient’s neurological function continued to decline, and she ultimately succumbed to the disease within months, a course typical of advanced PML with profound immunosuppression [13].

## Conclusions

This case underscores the diagnostic challenges of progressive multifocal leukoencephalopathy (PML), particularly when it presents with subacute onset and progression of neurological deficits in individuals without a known history of immunosuppression. The discordance between clinical and radiological findings prompted further multidisciplinary evaluation, ultimately revealing undiagnosed advanced HIV infection and JC virus reactivation as the underlying cause. Despite the timely initiation of antiretroviral therapy, the patient’s condition deteriorated due to PML-associated immune reconstitution inflammatory syndrome. This highlights that early recognition and ART initiation may improve outcomes, although neurological recovery remains limited in many cases.
